# Interaction-focused music therapy with cancer-affected children and their significant others: a randomized controlled feasibility study with subsequent intervention (INMUT)

**DOI:** 10.1186/s40814-024-01490-8

**Published:** 2024-05-28

**Authors:** Constance Boyde, Bettina Berger, Alfred Längler, Lutz Neugebauer, Stine Lindahl Jacobsen, Rachel Swanick, Christine Gaebel, Dominik Schneider, Benedikt Bernbeck, Michael Paulussen, Thomas Ostermann, Christina Hunger-Schoppe

**Affiliations:** 1 Faculty of Health, Departement of Psychology and Psychotherapy, Chair for Clinical Psychology and Psychotherapy III, Witten/Herdecke University, Alfred-Herrhausen-Strasse 50, Witten, 58455 Germany; 2 Department of Paediatrics and Adolescent Medicine, Community Hospital Herdecke, Gerhard-Kienle-Weg 4, Herdecke, 58313 Germany; 3 Interprofessional Graduate School of Integrative Medicine and Health Sciences (IGIM), Witten/Herdecke University, Alfred-Herrhausen-Strasse 50, Witten, 58455 Germany; 4 Faculty of Health, Department of Human Medicine, Chair for Medical Theory, Integrative and Anthroposophic Medicine, Witten/Herdecke University, Gerhard-Kienle-Weg 4, Herdecke, 58313 Germany; 5 Faculty of Health, Department of Human Medicine, Chair for Paediatrics, Witten/Herdecke University, Dr. Friedrich-Steiner-Strasse 5, Datteln, 45711 Germany; 6 Nordoff/Robbins Centre for Music Therapy Witten, Ruhrstrasse 70, Witten, 58452 Germany; 7 Department of Communication and Psychology, Faculty of Sciences and Humanities, Aalborg University, Rendsburggade 14, Aalborg, 9000 Denmark; 8 Chroma Therapies Ltd., Overross House, Ross Park, Ross-On-Wye, Herefordshire, HR9 7US UK; 9 Institute of Medical Psychology, University Hospital Heidelberg, Ruprecht-Karls University Heidelberg, Bergheimer Str. 20, Heidelberg, Germany; 10 Clinic for Paediatrics and Adolescent Medicine, Clinic Centre Dortmund, Beurhausstrasse 40, Dortmund, 44137 Germany; 11 Vestic Children’s Hospital, Dr. Friedrich-Steiner-Strasse 5, Datteln, 45711 Germany; 12 Faculty of Health, Department of Psychology and Psychotherapy, Witten/Herdecke University, Alfred-Herrhausen-Strasse 50, Witten, 58455 Germany

**Keywords:** Music therapy, Paediatric oncology, Assessment of parent–child interaction (APCI), Feasibility randomized controlled trial, Family, Significant other, Systemic functioning, Psychological functioning, Psychosocial burden

## Abstract

**Background:**

Paediatric oncology/haematology patients and their families are confronted with a life-threatening situation for which music therapy can be a cross-linguistic field of action. The creative act of making music together offers the possibility to strengthen competences and make conflicts tangible. Besides its complementing of evidence-based biomedical care, there is little research on the feasibility and efficacy of interactive music therapy including the diagnosed child and their significant others.

**Methods:**

We conducted an assessor blind, prospective, multicentric feasibility randomized controlled trial (RCT) with subsequent intervention. Including overall 52 child-significant other dyads, INMUT investigates interaction-focused music therapy with cancer-affected children and their significant others (INMUT-KB; *n* = 21) compared to music therapy only with the child (MUT-K; *n* = 21) and a wait-list group (WLG; *n* = 10). The measurement points include the screening for a cancer diagnosis, psychometric baseline (pre-T1), initial assessment (T1/T2), music therapy sessions (T3–T9), final assessment (T10), final psychometric evaluation (post-T10), and 3-month follow-up (cat-T11). *Feasibility and acceptability* of the (1) research methodology, (2) intervention and (3) estimation of effect sizes will be assessed using qualitative and quantitative data. The *proposed primary outcome* includes the parent–child interaction (APCI), and the *proposed secondary outcomes* refer to subjective goal achievement (GAS), quality of life (KINDL), system-related functional level (EXIS), psychosocial stress (BAS), psychosomatic complaints (SCL-9k), and resources (WIRF). We plan to investigate the efficacy of INMUT-KB and MUT-K post-intervention (post-T10) within the RCT design and at 3-month follow-up (cat-T11).

**Discussion:**

This study will provide insights into the feasibility of INMUT and the final sample needed for a confirmatory RCT. We will reflect on successfully implemented study procedures and, if necessary, provide recommendations for changes considering the design, procedures, measures, and statistical analyses. The discussion will conclude with an evaluation whether a confirmatory RCT is worth the investment of future resources, including the calculated number of child-significant other dyads needed based on the efficacy trends derived from this feasibility study.

**Trial registration:**

ClinicalTrials.gov: NCT05534282; date of registration: June 23, 2022.

## Background

Music therapy has asserted itself as an interdisciplinary field of psychosocial care in paediatric oncology [1]. Since 2004, it is part of the professional profile of the Psychosocial Working Group for Paediatric Oncology and Haematology (PSAPOH) [2] and was anchored in the S3 guidelines of the PSAPOH in 2008 [3]. The inclusion of significant others in therapy is explicitly recommended in these guidelines. “Significant others” is a psychological term which has been adopted [4–7] to describe people who are close to the cancer-affected child, bearing responsibility and providing support, such as parents, grandparents, foster parents, godparents, and siblings. The practical implementation of the inclusion recommendation however is not satisfactory. Only about half of the German-speaking clinics provide music therapy [8]. Up to now, the legal health care system in Germany covers inpatient music therapy for single patients in an individual setting. Outpatient music therapy is not reimbursed by the health insurance companies. Although significant others often also appear to be clinically affected [9], they are neglected. Consequently, there is a large gap in the psychosocial support of children with cancer and their social environment [10].

### State of research

We conducted a systematic literature search on PubMed and Cochrane databases in German and English with the time restriction 2011 to 2023. In the PubMed database, 52 literature references were found, 30 sources were not included due to irrelevance, and 22 articles were preselected because they matched at least 3 of 4 search categories. Of those, 15 articles were examined in more detail because they matched all search parameters. The sample included two scoping reviews, seven exploratory practice reviews, five quantitative studies such as randomized controlled trials (RCTs) and a single case design pilot study, and one qualitative study (Table 1).

1. *Scoping reviews*: The results showed that music therapy can address the needs of paediatric oncology patients and families and optimize their care [11]. Music therapy is suitable for promoting self-esteem; improving physical, emotional, and cognitive aspects related to the disease; and, to a lesser extent, alleviating physiological symptoms. Music therapy interventions are generally well received, not only by children and adolescents with cancer but also by their families and health professionals. Nevertheless, several gaps were identified in the studies reviewed, including a lack of specificity in terms of the outcomes achieved or the music therapy intervention methods used [12].
2. *Exploratory practice reviews*: The importance of music in the lives of children and their parents in paediatric oncology is intensely discussed [13]. Music provides an experience of competence and interactive affect regulation [14] and thus is suggested to be a measure of quality improvement for the future growth of the music therapy profession [15]. A multicentric survey study highlights the importance of communication and collaboration between music therapists and the multidisciplinary care team using a model of family-centred care that actively involves parents and caregivers in music interventions, treatment planning, and care delivery [16]. In a feasibility and acceptability report, patients, parents, and family members describe active music-making as a coping mechanism for pain, discomfort, stress, anxiety, and boredom. Music-making increases the children’s and significant others’ relaxation and sleep quality and is associated with pleasure and bonding [17]. Two articles report parents’ perspectives [18] and adolescents’ perspectives [19] on music-induced mechanisms of action in relation to positive coping, resilience, social support, and family function in an RCT stemming from the research team around Sheri L. Robb (cf. [10]).
3. *Quantitative studies*: In their RCT, Robb et al. report improvement in health outcomes of courageous coping, social integration, and family environment during high-risk cancer treatment using therapeutic music video interventions [10]. Another RCT and a single case design pilot study from Robb et al. point out the positive effects of active music interventions on multiple biomarkers to improve understanding of dose–response effects [20, 21], as well as treatment fidelity in music-based play interventions for young children with cancer and their parents [22]. In addition, a pilot RCT by Robb et al. investigated the feasibility and acceptability of parent-delivered active music engagement. This intervention was successful in providing emotional relief to children but was not well received by parents due to the unfavourably planned intervention content [23].
4. *Qualitative studies*: The qualitative study investigates the effects of musical training on the psychological well-being and quality of life of brain tumour survivor children, their parents, and caregivers. Although positive effects were reported, the factors that stimulated the efficacy are still unknown [24].

**Table 1:** Systematic keyword-based literature search

**No**			**Searches**	**Results**
**PubMed from 2010 to 2023; searched 12.01.2023**				
1		All fields	“music therapy” OR “music intervention” OR “music based”	13.436
2	AND	All fields	“oncology” OR “cancer”	803
3	AND	All fields	“children” OR “paediatric”	129
4	AND	All fields	“famil*” OR “parents”	52
**Cochrane from 2010 to 2023; searched 12.01.2023**				
1		All fields	“music therapy” OR “music intervention” OR “music based”	2.745
2	AND	All fields	“oncology” OR “cancer”	279
3	AND	All fields	“children” OR “paediatric”	2
4	AND	All fields	“famil*” OR “parents”	0

Though these publications strongly recommend the inclusion of significant others in the practice and research of music therapy, there is no RCT in interactive music therapy focusing on effects in the child-significant other interaction.

### Epidemiology

Although the probability of a child to fall ill with cancer in the first 18 years of life is only 0.3%, oncological and haematological diseases are the second most common cause of death in children and adolescents [25]. In Germany, about 2200 young people under the age of 18 are affected every year; the Society for Paediatric Oncology and Haematology [25] speaks of an incidence of around 170 new cases per 1 million children in this age group. The most common types of cancer in children and adolescents are leukaemia (about 30%), followed by tumours of the central nervous system (about 24%), lymphomas (about 14%), soft tissue sarcomas (5.7%), neuroblastomas (5.5%), and nephroblastomas (about 4.2%) [25].

### Clinical picture

According to a definition of the PSAPOH paediatric oncology patients are confined in an existential stress situation. They oscillate with their families in a field of tension caused by extreme experiences. Following the shock of a life-threatening diagnosis, everyday life starts to break down and is soon to be determined by hospital stays including invasive medical interventions and strict therapy plans as well as social restrictions in the home environment. In addition to physical effects, children with cancer and their relatives are confronted with emotional distress caused by worries, fears, feelings of helplessness, isolation, and the loss of control and autonomy. Within these affected social systems, mainly the family, individual and systemic coping strategies must be developed which, in conjunction with existing interaction patterns, have a great impact on how these stressful situations are dealt with [2].

### Music therapy

Although music therapy is still far from being an integrated part of multi-professional paediatric clinic teams in German-speaking countries, it is recommended as an integral part of the inpatient psychosocial care for children and adolescents with cancer [26, 27]. In addition to more linguistically oriented forms of therapy and counselling, it offers the possibility to express, experience, and reflect on an emotional-intuitive level through musical creation [2]. The identity-building act of self-experience creates a space in which the children and adolescents can confront the internal and external challenges of their current existential situation. They deal with physiological, psychological, and social conditions in musical improvisation [28]. Individual and age-specific developmental phases as well as developmental-psychological processing strategies are considered. In its professional profile of art and music therapists, the PSAPOH recommends music therapy for (1) patients with physical stress criteria, such as drastic changes in body image, severe courses of illness, painful medical interventions, recurrences, prolonged hospital stays, isolation (e.g,. in bone marrow transplants), immobility due to treatment, speech impairments, motor and sensory disabilities, and poor prognosis; (2) patients with psychological stress criteria, such as severe anxiety, processing problems (e.g,. regression, severe withdrawal, aggressive behaviour), compliance problems, and particular psychological and vegetative reactions (e.g., depressive symptoms, perceptual disorders, dissociation, decompensation); (3) patients in palliative therapy; (4) patients with existing language barriers; and (5) outpatients and follow-up care in the case of permanent changes in body image or physical disabilities, reintegration difficulties, behavioural problems occurring after the end of intensive therapy, the child’s wish to continue the accompaniment, identity problems, traumatic experiences and their processing, stigmatization, and difficulties in coping with the disease and mourning [2].

### Efficacy of music therapy

In the health technology assessment (HTA) report on music therapy published in 2019, the German Institute for Quality and Efficiency in Health Care [1] describes indications and evidence for a short-term efficacy of music therapy in relation to psychological endpoints such as fatigue, mood swings, anxiety, and health-related quality of life [1]. In the studies we evaluated and selected for this purpose, evidence was found that music-based interventions have positive effects on psychological well-being [17, 24], competence, resilience, and interactive affect regulation [14]. Positive effects have been found in relation to cognitive and physiological aspects [12], as indicated by biomarkers in form of cortisol and immune function measurements [20, 21] and measurement of heart rates, respiratory rates, and blood pressure [29]. Music therapy has also been claimed to support family functioning and social integration [10, 16, 18, 19, 30].

### Pilot study

RCTs are considered the most rigorous standard of evidence-based research. They are often complex, time-consuming, and expensive. Before implementing a confirmatory RCT, a feasibility RCT should be conducted that replicates all the essential elements of the planned larger trial [31]. This study is the first feasibility RCT, and the first RCT in general, which focuses not only on the affected children but also on their equally important significant others. INMUT is also the first feasibility RCT to use both the blind observer-based music therapy assessment of parent–child interaction (APCI) [32, 33] in paediatric oncology and a self-report questionnaires for the child as well as the significant other addressing quality of life, systemic and psychological functioning, psychosocial burden, resources, and goal attainment. The aim is to investigate the needed conditions for a subsequent confirmatory RCT which could save costs by carrying out the interventions more efficiently in the future with a well-powered study sample [34].

### Aims and objectives

The main focus of this assessor-blind, prospective, multicentric feasibility RCT is the implementation of the necessary recruitment procedures, for the inclusion of child-significant other dyads (INMUT-KB), the music therapy as usual with only the child (MUT-K), and a wait-list group (WLG). Further objectives were considering procedures for data analyses, the exploration of potential effects and the calculation of child-significant other dyads needed for a subsequent confirmatory RCT. Particularly in vulnerable patient groups such as the clientele described here, a careful estimation of the case numbers is necessary in order to be able to validate the primary and secondary effects in the confirmatory RCT, without spending resources on a unnecessarily large sample size that bears the risks of bias and noise [34, 35]. For that purpose, we will descriptively and exploratively investigate the following research questions (RQ).

1. Feasibility and acceptability of research methodology
  *RQ1.1*: Is it possible to recruit and randomize enough child-significant other dyads according to the study protocol? What prevents inclusion, and what causes dropouts?
  *RQ1.2*: Is it possible to recruit enough music therapists with adequate qualifications at the co-operating clinics? Can supervisors be found to provide the music therapists with professional support?
  *RQ1.3*: Have the music therapists been successfully certified as raters of the assessment of parent–child interaction (APCI)? Is a blinded implementation of the APCI feasible in the study? Can supervisors be found to provide the APCI raters with professional support?

2. Feasibility and acceptability of the interventions
  *RQ2.1*: Do child-significant other dyads accept and adhere to the intervention as measured by APCI ratings (T1, T2, and T10)?
  *RQ2.2*: How do the APCI raters rate the implementation of the assessment (T1, T2, and T10) and their own manual adherence?
  *RQ2.3*: Do the child-significant other dyads accept and adhere to the music therapy interventions (T3–T9)? Can the individual conditions of the sample in the three study arms (INMUT-KB), (MUT-K), and (WLG) be met?
  *RQ2.4*: How do the music therapists assess the implementation of the music therapy interventions (T3–T9) and their own manual adherence?
  *RQ2.5*: What additional benefits and barriers (T1–T10) result from the significant other’s participation in the child’s music therapy session? How do they perceive their participation in this research study? Do they report adverse events during the study period, and if yes, which ones?

3. Estimation of effect sizes
  *RQ3.1*: How likely is it that potential between-group effects of the interventions by INMUT-KB compared to MUT-K are due to the involvement of the significant others in the music therapy interaction?
  *RQ3.2*: What is the estimated within-subjects effect size and 95% CI for change in the proposed primary outcome, the APCI score, which encompasses measures of mutual attunement, nonverbal communication, and parental emotional response from baseline to the end of music therapy?
  *RQ3.3*: What is the estimated within-subjects effect size and 95% CI for change in the proposed secondary outcome, i.e. subjective goal achievement, quality of life, system-related functional level, psychosocial stress, psychosomatic complaints, and resources from baseline to the end of music therapy?

## Methods

### Design

This study is planned as an assessor blind, prospective, multicentric feasibility RCT with subsequent intervention. A total of 52 clients will be randomized to either the newly developed music therapy (INMUT-KB; *n* = 21), the music therapy as usual (MUT-K; *n* = 21), or the wait-list group (WLG, *n* = 10). The intervention outcome will be measured at three assessment points: baseline; post-intervention (primary endpoint), and 3-month follow-up.

### Inclusion and exclusion criteria

#### Child-significant other dyad

The study will include *children* with (1) an oncological/haematological disease in acute inpatient treatment with chemotherapy and/or radiotherapy, (2) age range between 5 and 13 years, and (3) adequate language skills for study-related self-reporting. Exclusion criteria encompass the following: (1) a serious physical comorbidity with impairment of brain-organic functions, (2) auditory impairment, (3) BMI < 14, (4) insufficient language skills, and (5) withdrawal of informed consent for study participation. Inclusion criteria of *significant others* refer to the following: (1) their role as a significant other (e.g. mother, father, aunt, caregiver) and (2) sufficient language skills for study-related self-reporting. Exclusion results if (1) informed consent for study participation is withdrawn.

#### Therapists

Inclusion criteria for music therapists encompass a state-recognized degree from a music therapy institution certified by the German Music Therapy Association (DMtG) and professional experience in a clinical context, particularly in the fields of paediatrics and oncology/haematology. Additionally, for the rating of the child-significant other dyads in the context of the music therapy assessment, they must be certified for the APCI [32, 33].

### Recruitment

#### Patients and significant others

Recruitment will start in 2023 and end when 52 child-significant other dyads are included which is expected to be in autumn 2024. The medical management and interprofessional team of the cooperating clinics, i.e., the Community Hospital Herdecke, Clinic Center Dortmund, and Vestic Children’s Clinic Datteln, will call attention of the cancer-affected children and their significant others towards this study within the inpatient setting. Prior to that, we will have informed the medical management and the interprofessional team about the study and will have distributed flyers and study information to them. All patients will present themselves to the study team, and no patients will be referred.

#### Therapists

Recruitment started in 2021, and we already included 9 music therapists with a state-recognized degree from a music therapy institution certified by the German Music Therapy Association (DMtG). The certification process for the primary outcome, i.e. the assessment of parent–child interaction (APCI) [32, 33], began in November 2021. A 3-day training was followed by a trial phase with five non-clinical child-significant other dyads for each music therapist. The implementation of the assessments, embedding the APCI survey in the statistical programme, and the writing of the APCI reports were accompanied and reviewed individually by the APCI trainer and concluded with a joint meeting. Certification took place after completion of all supervisions by the APCI trainer in August 2022.

### Study procedures

**Fig. 1:**
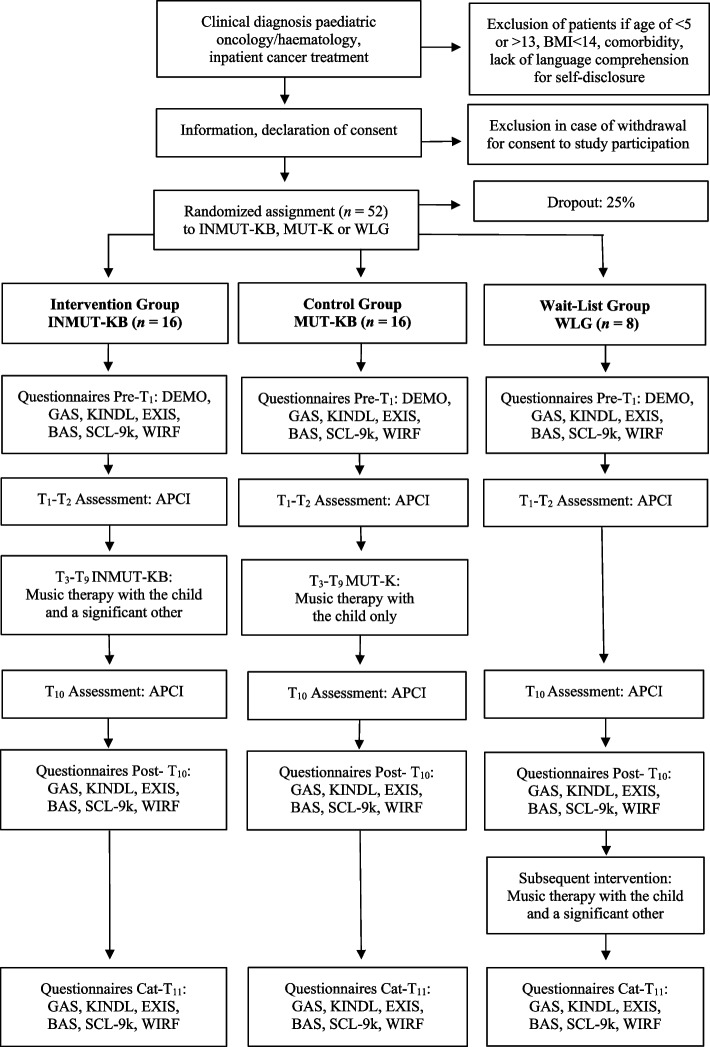
Design, assessments and patient flow (CONSORT chart). Note: APCI Assessment of Parent-Child Interaction, DEMO Demographic Data Collection, KINDL Children and Young People Quality of Life, EXIS Experience in Social Systems, BAS Burden Assessment Scale, SCL-9k Symptom Checklist, WIRF Witten Resources Questionnaire

Design, assessments and patient flow can be found in Fig. 1, assessment measures and application plan can be found in Table 2.

**Table 2:** Assessment measures and application plan

**Purpose**	**Perspective**	**Domain**	**Instrument**	**Screening**	**Before the start of T1**	**1st & 2nd hour of therapy**	**3rd to 9th hour of therapy**	**10th hour of therapy**	**After therapy T10**	**3 months after ending of therapy T10**
					**Pre-T1**	**T1–T2**	**T3–T9**	**T10**	**Post-T10**	**Cat-T11**
Screening	MD	Clinical diagnosis, Cancer treatment	CD	X						
Demographic	P, SO, T, A	Demographic data	DEMO		X					
Primary Outcome	A	Mutual attunement	APCI			X		X		
	A	Nonverbal communication	APCI			X		X		
	A	Emotional parental response	APCI			X		X		
Secondary Outcome	P, SO	Subjective goal achievement	GAS		X				X	X
	P, SO	Quality of life	KINDL		X				X	X
	P, SO	System-related functional level	EXIS		X				X	X
	SO	Psychosocial burden	BAS		X				X	X
	SO	Psychosomatic complaints	SCL-9k		X				X	X
	SO	Resources	WIRF		X				X	X
Process	P, SO, T, A	Significant moments	SM			X	X	X		
Quality	T, A	Manual adherence	AC			X	X	X		

*P* Patient, *SO* Significant Other, *A* Assessor, *T* Therapist, *MD* Medical Doctor, *CD* Clinical Diagnosis, *APCI* Assessment of Parent-Child Interaction, *DEMO* Demographic Data, *KINDL* Children and Young People Quality of Life, *EXIS* Experience in Social Systems, *BAS* Burden Assessment Scale, *SCL-9 k* Symptom Checklist, *WIRF* Witten Resources Questionnaire, *GAS* Goal Attainment Scaling, *SM* Significant Moments, *AC* Adherence and Competence

#### Screening 

The medical management of the cooperating clinics will provide the screening of inclusion criteria. If the inclusion criteria are met, the child and significant other will receive the recruitment information. The information material and consent forms are specially designed for children; if they are not yet able to write themselves, their parents or guardians do this on their behalf. The child’s consent is a prerequisite for the realization of each session. They can refuse to participate at any time. After having obtained informed consent, the child-significant other dyad will be randomly assigned to one of the three study arms and informed about their assignment. Together with the interprofessional team of the responsible clinic, it will be decided whether the music therapy will take place on an outpatient basis, i.e. the Nordoff/Robbins Music Therapy Centre, Witten, or on an inpatient basis in the clinics, either in the music therapy unit or on the hospital wards.

#### Baseline (pre-T1, T1/T2)

The paper–pencil baseline survey will be completed by all children (approx. 10 to 15 min) and significant others (approx. 20 to 30 min) (pre-T1), i.e. up to a maximum of 3 days prior to the APCI with its two manualized entry music therapy sessions (approx. 25 min each) (T1/T2). The APCI music therapy sessions will be video-recorded and evaluated using the APCI online programme. APCI raters are blinded and will give information about possible significant moments in T1/T2 and about their adherence to the APCI manual based on self-evaluation. The child and significant other will also provide information about their significant moments if present. Two independent student raters will rate one of the two APCI sessions for APCI manual adherence.

#### Therapy (T3 bis T9)

The music therapy interventions will be provided in seven sessions (T3 to T9). They will last about 20 to 45 min, will be video-recorded, and descriptively documented by the music therapists. At the end of each music therapy session, the child, significant other, and music therapist will give information about significant moments. The music therapists will self-evaluate their adherence to the INMUT-KB or MUT-K manual. Two independent student raters will rate two of the seven intervention sessions for INMUT-KB or MUT-K manual adherence.

#### Outcome (T10, post-T10)

The final APCI session (T10) will last about 25 min, will be video-recorded, and evaluated using the APCI online programme. APCI raters are blinded and will give information about significant moments in T10 and a self-evaluation on their adherence to the APCI manual. The child and significant other will provide information about their significant moments. Two independent student raters will rate the final APCI session for APCI manual adherence.

The paper–pencil outcome survey will be completed by all children (approx. 10 to 15 min) and significant others (approx. 20 to 30 min) (post-T10), i.e. up to a maximum of 24 h after the final APCI.

#### Follow-up (cat-T11)

A paper–pencil follow-up survey will be completed 3 months after the last APCI was carried out by all children (approx. 10 to 15 min) and significant others (approx. 20 to 30 min) (cat-T11).

### Randomization

#### Treatment allocation

We will use block randomization of 10 to 12 child-significant other dyads each. From the total of 52 child-significant other dyads to be included, 21 dyads will be randomly assigned to the INMUT-KB, 21 dyads to MUT-K, and 10 dyads to WLG. The randomization plan will be carried out by an independent research assistant at the Heidelberg University Hospital, who is not otherwise involved in the study, using the statistical software R version 4.2.1 [36, 37]. The randomized allocation of the child-significant other dyads into the three study arms will be performed after finalization of the screening. The allocation will be carried out by means of sealed envelopes (“sealed envelope method”), which will be opened in the presence of the child-significant other dyads together with a study staff member.

### Blinding and Allegiance

#### Blinding

The APCI sessions at baseline (T1/T2) and end of therapy (T10), indicating the primary endpoint of this feasibility RCTs, will be blindly conducted by APCI raters. The music therapists will know the assignment as they will also guide the INMUT-KB and MUT-K (T3 to T9). APCI raters will not be the music therapists who are otherwise working with the child-significant other dyads. The included child-significant other dyads will be informed about their assignment and the general aims of the study, but not about specific hypotheses of any endpoint.

#### Allegiance

Researchers’ and therapists’ preference for a particular treatment can lead to bias in the outcome data [38]. To avoid such bias, we will use multiple music therapists in the role of APCI assessors and/or INMUT-KB and MUT-K music therapists. They come from different music therapy traditions and are open to use their method but must be loyal to the INMUT-KB and MUT-K music therapy manual, respectively. They will all receive equal support and contribute equal numbers of hours and child-significant other dyads to the project [39].

### Music therapy intervention

#### Manual

The process-oriented music therapy interventions are methodologically oriented towards Nordoff/Robbins, Neugebauer, and Aldridge [40–42] but take into account the different practical experiences of the respective therapists. The process orientation serves the stimulation of creativity in music therapy and offers space for (1) the use of melodic, harmonic, and rhythmic instruments that can be played and freely chosen without prior knowledge; (2) individual session lengths adapted to the needs of the child and significant other, if present; (3) objectives tailored to the participant(s); (4) taking up topics of the participant(s) and integrating them into the interventions; (5) situational decisions about both active and receptive offers; (6) promoting relationship work in musical improvisation; and (7) linking to individual resources of the child and significant other, if present. The focus is the relationship work in musical improvisation. While this develops between child and music therapist in the individual sessions (MUT-K), during the multi-person setting, the focus is on the musical togetherness in the child-significant other dyad (INMUT-KB). The goal is that the same significant other attends all music therapy interventions. In exceptional cases, a change of significant others is permitted. For a better reflection, all music therapy sessions will be video-recorded. The therapists offer to watch the recordings together with the families to formulate significant moments. The overall process of the music therapy intervention is presented to the interprofessional team of our cooperating clinics and the research team in a descriptive report written by the music therapists.

#### Adherence

The therapists will self-evaluate their adherence to the music therapy manuals (INMUT-KB, MUT-K) after each session. In addition, two independent student raters will rate selected sessions for manual adherence.

### Measures

As the purpose of this study is to explore the feasibility of a fully powered RCT, we will use different measures, including both qualitative information and quantitative data.

1. Feasibility and acceptability of research methodology
  *RQ1.1*: The feasibility analysis of the protocol implementation focusses on the overall recruitment target of the child-significant other dyads (*n* = 52), as well as the specific recruitment target for each study-arm INMUT-KB (*n* = 21), MUT-K (*n* = 21), and WLG (*n* = 10). The study considers the estimation of the total sample to be recruited, the overall dropout rate, and the differentiated dropout rate within the study arms at all measurement times. Data will be collected by observing the participant flow: Counting the number of study participants who (1) are recommended for inclusion by the medical team; (2) are contacted by the study staff to provide information about the feasibility RCT; (3) give their consent to study participation; (4) will be included into the study; (5) drop out before the baseline survey (pre-T1); (6) complete the baseline survey (pre-T1); (7) drop out before or during the baseline APCI ratings (T1/T2); (8) complete the baseline APCI ratings (T1/T2); (9) drop out before or during the INMUT-KB, MUT-K, and WLG (T3–T9); (10) complete INMUT-KB, MUT-K, and WLG (T3–T9); (11) drop out before the final APCI rating (T10); (12) complete the final APCI rating (T10); (13) drop out before the outcome survey (post-T10); (14) complete the outcome survey (post-T10); (15) drop out before the 3-month follow-up survey (cat-T11); and (16) complete the 3-month follow-up survey (cat-T11). We will describe the causes for dropout and calculate the retention rate for the three study arms separately and together. Success is defined as attaining 100% and retaining 75% of the planned sample.
  *RQ1.2*: The feasibility of this RCT depends on the recruitment of a sufficient number of music therapists. We expect around five to six cases per music therapist, so that successful recruitment will involve at least seven to eight music therapists. For professional support, every third session will be supervised; based on the case numbers, we are planning 108 supervised sessions.
  *RQ1.3*: Certification to use the APCI was started by seven music therapists and successfully completed by five. The APCI raters were trained in the practical application of the APCI manual, video analysis, data collection, and statistical analysis. Experience-based receptive interviews [43] with APCI raters will gather information about the circumstances that contribute to successful APCI implementation. The success of APCI blinding will also be evaluated in receptive interviews. For professional support, every third APCI session will be supervised; based on the case numbers, we are planning 49 supervised sessions.

2. Feasibility and acceptability of the interventions
  *RQ2.1*: The feasibility and acceptance of the APCI ratings (T1, T2, and T10) by study families are assessed by analysing the therapists’ final APCI descriptions.
  *RQ2.2*: How well APCI raters implement the APCI ratings (T1, T2, and T10) is assessed by their self-assessment after each APCI session using an APCI manual adherence scale (range: 0 to 100). In addition, randomly selected first or second sessions (T1, T2) and final sessions (T10) are evaluated by two independent observers.
  *RQ2.3*: The feasibility and acceptance of the music therapy interventions (T3–T9) by study families are assessed on a visual analogue scale (range: 0 to 100), complemented by experience-based reports in receptive interviews [43] conducted with the study families during the study period. In particular, the needs of the child-significant other dyads are analysed with a special focus on the involvement of significant others in the music therapy process. Compliance with the INMUT-KB and MUT-K conditions by the children and significant others is assessed based on the percentage of attempted and completed music therapy sessions. Compliance with the WLG is assessed according to whether alternative music therapy interventions outside the trial were used during the waiting period.
  *RQ2.4*: How well music therapists implement the music therapy interventions (T3–T9) is assessed by their self-assessment after each session using an intervention manual adherence scale (range: 0 to 100). In addition, every third session is evaluated by two independent observers.
  *RQ2.5*: Additional identified benefits and barriers of INMUT-KB, MUT-K, and WLG will be monitored throughout the study and considered in relation to intervention safety and potential adverse outcomes, in addition to experience-based reports in receptive interviews [43]. For any undesirable psychological and/or somatic effects during or after the music therapy interventions, psychological psychotherapists at the Centre for Mental Health and Psychotherapy (ZPP) at the UW/H, directed by Prof. Dr. Christina Hunger-Schoppe, will be available.

3. Estimation of effect sizes
  *RQ3.1*: The potential effect of including significant others is measured using the quantitative and qualitative data of the APCI ratings, including its three subscales and the final score, at baseline (T1 and T2) and final assessment (T10)*.*
  *RQ3.2*: The estimation of effect size and 95% CI for change will concentrate on the proposed primary outcome, i.e. APCI subscores of mutual attunement, nonverbal communication, and parental emotional response from baseline to the end of music therapy.
  *RQ3.3*: The estimation of effect size and 95% CI for change will concentrate on the proposed secondary outcome, i.e. subjective goal achievement, quality of life, system-related functional level, psychosocial stress, psychosomatic complaints, and resources from baseline to the end of music therapy.

#### Proposed primary outcome measure

The *assessment of parent–child interaction (APCI)* [32, 33] was developed for at-risk families and is being used for the first time in an RCT to evaluate music therapy interaction processes in paediatric oncology. The APCI assesses the child-significant other dyad interaction based on three dimensions: mutual attunement, nonverbal communication, and emotional parental response. The goal is that the same significant other attends all APCI sessions. In exceptional cases, a change of significant others is permitted. The training manual [32] includes theoretical considerations, state-of-the-art literature considering the APCI, and information on how to plan, analyse, and report the collected information. The assessment sessions follow a manualized procedure: (1) welcome and exploration of the room and instruments; (2) the sequence of four different practice sessions, which are first carried out together with the therapists and subsequently by the child-significant other dyad alone; and (3) the farewell. The four sequences consist of (Ex1) playing a crescendo and decrescendo together, (Ex2) taking turns in making music and handing over the musical action, (Ex3) mutual leading and following in music, and (Ex4) free improvisation. Video evaluation is used to assess the three APCI dimensions which are transferred to the APCI online portal for the calculation of a total APCI score, subscores for each dimension, and further statistical analyses. The APCI total score is determined from 16 APCI profiles, which are made up of the following observations: (1) mutually attuned (M)/not mutually attuned (N), (2) clear nonverbal communication (C)/unclear nonverbal communication (U), (3) supportive parenting (S)/lack of parenting support (L), and (4) independent child (I)/dependent child (D). The APCI shows good to satisfactory interrater reliability and test-re-test reliability both in the overall score and in its subscores [33]. In our feasibility RCT, the raters of the APCI diagnostics were trained and certified by Rachel Swanick (Training Lead & Senior Clinical Therapist at Chroma Arts Therapies, UK) to use the APCI. During the study, they will be supervised by Rachel Swanick and Stine L. Jacobsen (Associate Professor of Music Therapy, Aalborg University, Denmark). Two independent student raters will rate one of the two first APCI session, and the final APCI session, for manual adherence.

#### Proposed secondary outcome measure

The *goal attainment scaling (GAS)* asks for the subjective goals of the children and significant others in free text. These are classified using the Bern Inventory of Therapy Goals (BIT-T) [44]: (1) coping with specific problems and symptoms, (2) interpersonal goals, (3) well-being, (4) existential issues, (5) personal growth, and (6) residual category. In [36] coding an extended sample of client treatment goals, the BIT-T proved to have a good interrater reliability, identified differences between diagnostic groups, and showed meaningful relations to standardized intake measures [45].

The quality of life will be assessed using the *KINDL Children and Adolescents Version, Oncology Module* [46, 47]. KINDL measures psychological well-being, social relationships, physical functioning, and daily activities within the last week using 40 items. The KINDL shows very good to satisfactory internal consistency and test-re-test reliability both in the overall score and regarding its dimensions. The questionnaire is a validated assessment for children and adolescents aged 3 to 17 and is accompanied by master’s students in psychology. It is carried out as independently as possible from the parents to allow a free, autonomous response.

The questionnaire on *Experience in Social Systems Questionnaire (EXIS)* [48] measures basic dimensions of systems functioning, i.e. accord, belonging, autonomy, and confidence in the future within the last 2 weeks with 12 items. The EXIS shows very good to good internal consistency, and test-re-test reliability, both in the overall value and regarding its dimensions. The EXIS children’s version has been developed with children from the age of 5 and is accompanied by master’s students in psychology. It is carried out as independently as possible from the parents to allow a free, autonomous response.

The *Burden Assessment Scale (BAS)* [4] uses 19 items to assess personal stress, feelings of guilt, the need to interrupt and postpone important matters, and a changed time perspective in connection with the symptoms displayed by the significant other of an ill person within the last 6 months. The BAS shows excellent internal consistency considering its overall score.

The short form of the *Symptom Checklist (SCL-9 k)* [49] measures impairment using nine items on somatization, obsessiveness, social insecurity, depressiveness, anxiety, aggressiveness, phobic anxiety, paranoid thinking, and psychoticism. The SCL-9 K is shown to have good internal consistency and test-re-test reliability on the Global Severity Index (GSI).

The *Witten Resources Questionnaire (WIRF)* [50] measures resources in the three domains of general coping, past difficult situations and current problems, using 12 items each. The WIRF shows good to satisfactory internal consistency in the overall score as well as regarding its dimensions. In our study, we will limit the WIRF to the domain assessing coping with current problems for economic reasons.

The *Development of Psychotherapist Common Core Questionnaire (DPCCQ)* [51–53] characterizes therapists on several subscales [54], of which we chose sociodemographic data, interpersonal style, relational skills, quality of therapists’ personal lives, and difficulties in practice to be suitable for this feasibility study. The scales have been found predictive of the therapeutic alliance and outcome.

Children and significant others will complete a brief *demographic measure*, including age, sex, education level, and whether they are employed.

### Sample size calculation

Assuming an estimated small between-group effect size (80% power, 5% significance level), we will need 344 child-significant other dyads in a confirmatory RCT for direct comparison of INMUT-KB and MUT-K [34]. This calculation grounds in the suggestion of at least 9% of the subsequently powered RCT sample to be seen in the prior feasibility RCT. Consequently, we will recruit at least 32 child-significant other dyads. Taking the WLG into account with an additional one-third of this sample, i.e. 10 child-significant other dyads, and a drop-out rate of, again, an additional 25% in each study arm, i.e. 10 child-significant other dyads in addition, the total sample size for our feasibility RCT will include 52 child-significant other dyads (intention to treat, ITT; INMUT-KB, *n* = 21; MUT-K, *n* = 21; WLG, *n* = 10). The number of participants expected to complete the trial encompasses 40 child-significant other dyads (per protocol, PP; INMUT-KB, *n* = 16; MUT-K, *n* = 16; WLG, *n* = 8).

### Statistical analyses

#### Feasibility and acceptability of research methodology

To analyse the feasibility and acceptability of the research methodology, we will calculate screening, recruitment, randomization, and drop-out rates considering the child and significant other, as well as missing data in the paper–pencil surveys at baseline (pre-T1), post intervention (post-T10), and at 3-month follow-up (cat-T11) (RQ1.1). We will calculate inclusion rates of intervention music therapists and APCI music therapists (RQ1.2, RQ1.3). The success of blind APCI ratings by music therapists, who are part of the study but not the music therapists of the child and significant other (RQ1.3), will be analysed performing Qualitative Content Analysis (QCA) [55] and/or Consensual Qualitative Research (CQR) [56, 57].

#### Feasibility and acceptability of the interventions

The *feasibility and acceptability of the APCI music therapy assessments (T1, T2, T10)* by the study families will be analysed performing QCA [55] and/or CQR [56, 57] on the therapists’ final APCI descriptions (RQ2.1). APCI music therapists’ adherence will be calculated as the percentage *(%)* of treatment components defined by the APCI manual across sessions that will be implemented as planned [58]. It will also be calculated using *Cohen’s kappa* considering the interrater reliability, based on data derived from randomly selected first or second sessions (T1, T2), and finals sessions (T10), evaluated by two independent observers (RQ2.2). To analyse the *feasibility and acceptability of the music therapy interventions (T3–T9)* by the study families, we will calculate mean scores (*M*), standard deviations (*SD*), and 95% confidence intervals (*CI*), in addition to percentages *(%)* considering data gathered on the visual analogue scale, the attempt and completion of the music therapy interventions, and the request for alternative music therapy interventions in the WLG. We will perform QCA [55] and/or CQR [56, 57] to analyse additional data collected on the basis of the study families’ experience-based reports [43] (RQ2.3). Music therapists’ adherence will be calculated as the percentage *(%)* of treatment components defined by the INMUT-KB or MUT-K manual across sessions that will be implemented as planned [58]. It will also be calculated using *Cohen’s kappa* considering the interrater reliability, based on data derived from the independent observers’ evaluation of every third music therapy session (RQ2.4). *Additional benefits and barriers* of INMUT-KB, MUT-K, and WLG will again be calculated by mean scores (*M*), standard deviations (*SD*), and 95% confidence intervals (*CI*), in addition to percentages *(%)*, with respect to intervention safety and potential adverse outcomes. Study families’ experience-based reports on these benefits and barriers will be analysed performing QCA [55] and/or CQR [56, 57] (RQ2.5).

#### Estimation of effect sizes

For *estimation of effect sizes*, and because child-significant other dyads treated by one music therapist (within-group) may be more like each other compared to those treated by another music therapists (within-group), a two-level linear regression analysis will be performed to account for potential clustering effects at higher levels (i.e. child-significant other dyads nested within therapists). The intraclass correlation (ICC) coefficient from the random intercept model with child-significant other dyad (Level 1) and therapist (Level 2) will be calculated for the proposed primary outcome (APCI) [59]. An ICC greater than zero will indicate a clustering effect, and any statistical analysis must be adjusted for these effects. All proposed outcomes will be analysed as intention to treat (ITT; *n* = 52) using mean scores (*M*), standard deviations (*SD*), and 95% *CI*. The ITT calculation will be regardless of whether the child-significant other dyads will violate the inclusion criteria, whether they complete the music therapy, or whether they will withdraw from the study due to protocol deviations (e.g. concurrent music therapy during the study period [60]). Missing values of less than 20% will be replaced with the conditional mean value of the subgroups (INMUT-KB, MUT-K, WLG). This will be compared to per-protocol analyses. We will use mixed-design ANOVAs (group: INMUT-KB, MUT-K, WLG; status: child-significant other dyad, child alone; time: baseline, post-intervention, 3-month follow-up), adjusting for baseline scores considering demography as well as outcome data. Because treatment outcome will be measured at three assessment points, within-group effects will be further analysed by comparisons between baseline and post-intervention (Contrast A) and by comparisons between post-intervention and 3-month follow-up (Contrast B) (RQ3.1). Effect sizes will be presented as partial eta-squared values (η^2^) and Cohen’s *d*, calculated as the difference between the means divided by the pooled standard deviation $(d=\frac{{x}_{1}^{2}-{x}_{1}^{2}}{\sqrt{{s}_{1}^{2}+{s}_{2}^{2}}/2})$. Classification of *effect sizes* will be as follows: η^2^ ≥ 0.010, small effect; η^2^ ≥ 0.060, medium effect; η^2^ ≥ 0.140, large effect; Cohen’s *d* 0.20, small effect; *d* ≥ 0.50, medium effect; and *d* ≥ 0.80, large effect [61] (RQ3.2, RQ3.3).

## Discussion

As far as we know, this is the first assessor-blind, prospective, multicentric feasibility RCT with subsequent intervention on interaction-based music therapy for children with cancer and their significant others. The already well-researched approaches to individual music therapy with only the child (MUT-K) will be compared to a multi-person setting including the child and his or her significant others (INMUT-KB). The study examines feasibility conditions to successfully recruit, include, and randomize the child-significant other dyads, as well as recruit, train, and supervise music therapist for the APCI diagnostics and the conduction of the INMUT-KB and MUT-K. INMUT investigates the implementation of additional study conditions such as the statistical analyses and finally the calculation of the number of child-significant other dyads needed for the subsequently planned confirmatory RCT.

### Innovative aspects

This trial will be the first feasibility RCT that directly compares a multi-person music therapy, including the child-significant other dyad (INMUT-KB), with a single child music therapy (MUT-K). INMUT is the first German RCT that uses a manualized assessment for child-significant other interaction (APCI), in addition to RCTs from countries like, e.g. Denmark [62, 63]. This assessment is adapted for the first time from work with at-risk families into a RCT working with families in paediatric oncology. The questionnaires on psychosomatic complaints (SCL-K-9), relative’s psychosocial burden (BAS), systemic functioning (EXIS), and resources for dealing with difficult situations (WIRF) are being used for the first time with important reference persons in paediatric oncology.

### Biases and limitations

This study is a feasibility RCT with interest in carefully planning the subsequently confirmatory RCT. All statistical analyses will be descriptive and exploratory with the aim of obtaining data that can be used to design a confirmatory RCT. Consequently, the main RCT is necessary before a confirmatory statement about the effectiveness of interaction-focused music therapy for children with cancer and their significant other can be made reliably. Though this is an assessor-blind study, children and their significant other, as well as the therapists, will be informed about which study arm they will be allocated to. This however is a naturalistic fact of “real-world delivery of care” ( [64] p. 6). We try to balance direct and indirect allegiance by conducting the music therapy sessions by equally educated music therapists from the different clinics on the group level and implementing high methodological quality that appears to buffer allegiance [39]. However, due to the small sample size, we will not be able to perfectly control for allegiance effects.

### Perspectives of a confirmatory trial

We aim to conduct a subsequent, assessor-blind, prospective, multicentric, confirmatory RCT comparing INMUT-KB with MUT-K and a WG in paediatric oncology. We will use the experience and results of our feasibility study to plan an RCT that is, again, lean in content and investigates, with a strong methodological rigour, the efficacy of interaction-based music therapy based on a powered and economically feasible number of child-significant other dyads. Furthermore, with the scientific presentation of music therapy effects, we are pursuing a strengthening of professional policy regarding the fight for approval of outpatient music therapy as a health insurance benefit in Germany. This would be a milestone for comprehensive and needs-oriented support for families with children suffering from cancer.

### Trial status

The trial is ongoing and is currently recruiting.

## Data Availability

All relevant data can be obtained by the first author.

## Abbreviations

APCI: Assessment of Parent–Child Interaction
BAS: Burden Assessment Scale
BIT-T: Bern Inventory of Therapy Goals
CSSRI-EU: Client Sociodemographic and Service Receipt Inventory
DEMO: Demographic Data
DGVM: German Society of Behavioral Medicine and Behavioral Modification
DMtG: German Music Therapy Association
EXIS: Experience in Social Systems
GAS: Goal Attainment Scaling
GPOH: Society for Paediatric Oncology and Haematology
GSI: Global Severity Index
HTA: Health Technology Assessment
ICC: Intra-Class Correlations
IFF: Internal Research Funding
IGIM: Interprofessional Graduate School of Integrative Medicine and Health Sciences
INMUT: Interaction-Focused Music Therapy
INMUT-KB: Interaction-focused Music Therapy with Child‐Significant Other Dyad
ITT: Intention-To-Treat Analyses
IQWiG: German Institute for Quality and Efficiency in Health Care
KINDL: Children and Young People Quality of Life
MUT-K: Music Therapy only with the Child
PSAPOH: Psychosocial Working Group for Paediatric Oncology and Haematology
RCT: Randomized Controlled Trial
SCL-9k: Symptom Checklist
SPSS: Statistical Package for Social Sciences
WIRF: Witten Resources Questionnaire
WLG: Wait-List Group
